# Carbon monoxide poisoning

**DOI:** 10.4103/0972-5229.68229

**Published:** 2010

**Authors:** H. Singh, S. Aggarwal

**Affiliations:** Intern, All India Institute of Medical Sciences, New Delhi; 1 Intern, University College of Medical Sciences, New Delhi

Dear Editor,

We read the article “Accidental carbon monoxide poisoning in our homes”[1] with great interest. The authors have done a commendable job to draw attention of doctors to think of CO poisoning from gas geysers as a potential cause of unexplained neurological collapse in bathrooms. We would like to add our comments and present our view on this.

As the practice of using LPG geysers is gaining popularity, the Government needs to create public awareness by appropriate use of mass media regarding the health complications attributed to CO poisoning caused by LPG geysers. Also, statutory warning regarding the proper place and ventilatory requirements for installation, potential medical problems and immediate action needed in case of suspected poisoning, should be mentioned on the geyser itself. This will greatly help to reduce the number of cases as well as encourage immediate medical attention seeking behavior of the user. We again appreciate the efforts of authors.
